# MLCD: A Unified Software Package for Cancer Diagnosis

**DOI:** 10.1200/CCI.19.00129

**Published:** 2020-03-27

**Authors:** Wenjun Wu, Beibin Li, Ezgi Mercan, Sachin Mehta, Jamen Bartlett, Donald L. Weaver, Joann G. Elmore, Linda G. Shapiro

**Affiliations:** ^1^ Department of Medical Education and Biomedical Informatics, University of Washington, Seattle, WA; ^2^ Paul G. Allen School of Computer Science and Engineering, University of Washington, Seattle, WA; ^3^ Department of Electrical and Computer Engineering, University of Washington, Seattle, WA; ^4^ University of Vermont Medical Center, Burlington, VT; ^5^ Department of Pathology and University of Vermont Cancer Center, Larner College of Medicine, University of Vermont, Burlington, VT; ^6^ Division of General Internal Medicine and Health Services Research, Department of Medicine, David Geffen School of Medicine at University of California, Los Angeles, Los Angeles, CA; ^7^ Craniofacial Center, Seattle Children’s Hospital, Seattle WA

## Abstract

**PURPOSE:**

Machine Learning Package for Cancer Diagnosis (MLCD) is the result of a National Institutes of Health/National Cancer Institute (NIH/NCI)-sponsored project for developing a unified software package from state-of-the-art breast cancer biopsy diagnosis and machine learning algorithms that can improve the quality of both clinical practice and ongoing research.

**METHODS:**

Whole-slide images of 240 well-characterized breast biopsy cases, initially assembled under R01 CA140560, were used for developing the algorithms and training the machine learning models. This software package is based on the methodology developed and published under our recent NIH/NCI-sponsored research grant (R01 CA172343) for finding regions of interest (ROIs) in whole-slide breast biopsy images, for segmenting ROIs into histopathologic tissue types and for using this segmentation in classifiers that can suggest final diagnoses.

**RESULT:**

The package provides an ROI detector for whole-slide images and modules for semantic segmentation into tissue classes and diagnostic classification into 4 classes (benign, atypia, ductal carcinoma in situ, invasive cancer) of the ROIs. It is available through the GitHub repository under the Massachusetts Institute of Technology license and will later be distributed with the Pathology Image Informatics Platform system. A Web page provides instructions for use.

**CONCLUSION:**

Our tools have the potential to provide help to other cancer researchers and, ultimately, to practicing physicians and will motivate future research in this field. This article describes the methodology behind the software development and gives sample outputs to guide those interested in using this package.

## INTRODUCTION

The long-term goal of the National Institutes of Health/National Institute of Cancer (NIH/NIC)–sponsored project, “A Unified Machine Learning Package for Cancer Diagnosis” (U01CA231782), which is part of the Information Technology for Cancer Research (ITCR) program, was the development of a unified software package for the diagnosis of cancer from whole-slide biopsy images. This article describes the resulting software, which leverages machine learning to automatically identify biomarkers, including a range of pathologic tissue types and possible cancer diagnostic categories, and can aid in improving the quality of both cancer research and clinical practice.

The field of pathology has been slow to move in an increasingly digitized age. However, US Food and Drug Administration authorization in April 2017 allowed the marketing of the first whole-slide imaging system for interpreting digital pathology slides from biopsy tissue samples,^1^ expanding our potential for research and opening opportunities in clinical practice for using digitized images in diagnostic assessment. NIH/NCI-sponsored research grants, including our own (R01 CA140560; R01 CA172343) have produced uniquely well-characterized biopsy images and methodology for finding regions of interest (ROIs),^2^ segmenting them into histopathologic tissue types,^3^ and using this segmentation as input to classifiers that suggest diagnoses.^4^ These methods are being converted into a unified Python software package, Machine Learning Package for Cancer Diagnosis (MLCD), with the corresponding modules available through the GitHub repository under the Massachusetts Institute of Technology license, to be distributed later with the Pathology Image Informatics Platform (PIIP) system developed by Martel et al.^5^ A demonstration is available on our Web page: https://cancertech.cs.washington.edu.

This article describes a software system suitable for a range of specialists, including cancer researchers studying large image data sets, practicing pathologists, experts who want a second opinion, and pathology residents who are learning the diagnostic process. There are many existing platforms, including those being developed in the ITCR program, that contain useful tools, but whose scopes differ from ours. HistomicsTK, developed by Cooper and Gutman et al,^6^ produces an image management platform and a set of generic image analysis algorithms for use in cancer biopsy image analysis. However, their breast cancer diagnostic tools lack the ROIs, segmentation, and diagnosis features offered in our software. Similarly, the XNAT platform, developed by Marcus et al,^7^ includes operational tools for data importing, exporting, archiving, organizing, sharing, visualizing, and reporting. The QuIP system, developed by Saltz et al,^8^ provides Web applications and analysis algorithms to load whole-slide images, detect nuclei in image tiles, and both visualize and explore the results. Saltz et al^9^ also has developed convolutional neural network (CNN) models for detecting patches containing lymphocytes and necrosis, which is related to our semantic segmentation module. QuIP differs in emphasis from our MLCD system, which is oriented toward fully automated whole-slide diagnosis. Our ROI detection is based on interest by examining pathologists, whereas theirs is based on tissue classification.

**CONTEXT**

**Key Objective**
Our goal was to create a software package that can automatically find, segment, and diagnose regions of interest in whole-slide biopsy images.
**Knowledge Generated**
A software package has been developed that uses classifiers to predict regions of interest to semantically segment them using a deep neural net and to classify them using a support vector machine into clinically relevant diagnostic categories.
**Relevance**
Our tools have the potential to help other cancer researchers and, with appropriate interfaces, support software-assisted interpretation of cancer biopsy slides by clinicians.

Our ROI tools are designed to help pathologists with their initial scanning of digital slides; these tools will be useful in studying the way pathologists identify diagnostic ROIs. Our tissue segmentation tools are designed to help cancer researchers study tissue type classification. Our diagnostic tools are designed to perform computer-aided diagnoses; researchers can use them to improve the quality of their studies, and in the future, practitioners can use them in their clinical work. For cancer researchers, most of our tools are generalizable from our breast cancer studies to other cancer types through retraining on new data sets. Thus, our tools will have a significant impact on both cancer research and clinical practice.

## METHODS

### Overview

**FIG 1.**
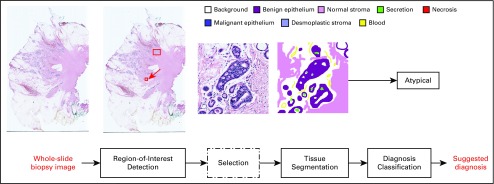
Overview of the proposed software system. There are 3 main modules: Region-of-Interest Detection, Tissue Segmentation, and Diagnosis. The flow begins with digital whole-slide hematoxylin and eosin (H&E)–stained images. The Region-of-Interest-Detection module is a classifier that selects regions within whole-slide images that are most likely to be useful in diagnosis and provides them to the user. The user can select one and send it to the next module; the Tissue Segmentation module uses a convolutional neural network to perform tissue segmentation. The resultant tissue map is used in the third module. The Diagnosis Classifier is a set of support vector machines that takes the region of interest and its tissue labels as input and produces the suggested diagnosis.

Our package is based on algorithms from the research articles mentioned in the Introduction and has 3 main modules as shown in Figure 1, Region-of-Interest Detection, Tissue Segmentation, and Diagnosis Classifiers. The workflow begins with digital whole-slide hematoxylin and eosin–stained (H&E) images whose average size is 90,000 × 70,000 pixels. The Region-of-Interest Detection module is a classifier that, based on training data from pathologists, selects regions within whole-slide images that are most likely to be useful in diagnosis and provides them to the user. The user can select one and send it to the next module, the Tissue Segmentation module, which uses a CNN that was trained on 58 ROI images whose pixels were manually labeled by an experienced pathologist with their tissue classes: background, benign epithelium, malignant epithelium, normal stroma, desmoplastic stroma, secretion, blood, and necrosis. The resultant segmentation, in the form of a labeled image, is for use in the third module: the Diagnosis Classifier. The Diagnosis Classifier is a set of support vector machines (SVMs) that takes feature vectors extracted from the labeled image and produces 1 of 4 different diagnoses: benign, atypia, ductal carcinoma in situ (DCIS), and invasive cancer. These classifiers were trained on data from our research projects. We will, in future work, also provide untrained classifiers for each step that can be trained by other researchers on their data.

### ROI Detection

Diagnostically relevant ROIs are represented using a visual bag-of-words model.^10^ This module computes a dictionary from small image patch clusters, which are its “words.” Then, the frequency of these words is used to describe larger image regions. Using frequency features, a classifier is trained to detect ROIs. The method consists of 2 major parts: (1) an ROIFeatureExtractor module, and (2) an ROIWindowClassifier module, both derived from algorithms developed by Mercan et al.^2^

The ROIFeatureExtractor takes in a whole-slide image and produces a feature vector that is used by the ROIWindowClassifier module. The input whole-slide image needs to be in a supported format, including TIFF, JPEG, BMP, or PNG.^1^ Other unsupported formats must first be converted. To achieve the optimal result, resolution of ×40 is recommended. Other resolutions are valid input, but the result might not be accurate because downsampling can cause information loss. The feature extractor consists of several internal submodules. First, a word constructor separates images into square patches called “words,” whose sizes are 120 × 120 pixels. Next, these visual words are converted from the RGB (red-green-blue) color space to the H&E space, using color deconvolution and stain normalization algorithms developed by Macenko et al.^11^ The visual words are also converted from the RGB color space into the L*a*b* color space using a nonlinear transformation.^12^ The L*a*b color space consists of 3 axes: R-G (red-green), Y-B (yellow-blue), and gray tone, and was the result of perceptual studies with humans conducted by He and Wang.^13^ Two histograms are then computed to describe the visual words: (1) a color histogram in the L*a*b* color space and (2) a texture histogram obtained from the Local Binary Pattern operator computed from the HE space. Next, the visual dictionary is constructed through applying k-means clustering to the histogram representations of the visual words, using both histograms as 1 large feature vector. Each cluster has a unique cluster number, and each word is assigned its cluster number. Thus, a larger image window (bag) can be represented as a histogram of the cluster numbers of its visual words. Finally, the SlidingWindowAnalysis^14^ module constructs the visual bags that cover these larger image windows whose sizes and overlap are 3,600- × 3,600-pixel windows, with 2,400-pixel overlap in both horizontal and vertical directions; the bags are represented by their histogram feature vectors.

In the ROIWindowClassifier module, the detection of diagnostically relevant ROIs is formulated as a binary classification problem where the samples are bags and the features are their word frequency histograms. During training, windows in ROIs were used as positive examples, and windows near, but not inside, the ROIs were used as negative samples to increase the discriminative ability of the classifier. This module is an SVM that takes in features computed in the ROIFeatureExtractor module to determine which image regions will be of interest to a diagnosing pathologist. The 2 outputs are a set of images for those regions and a visualization that shows them with red boxes.

### Tissue Segmentation

**FIG 2.**
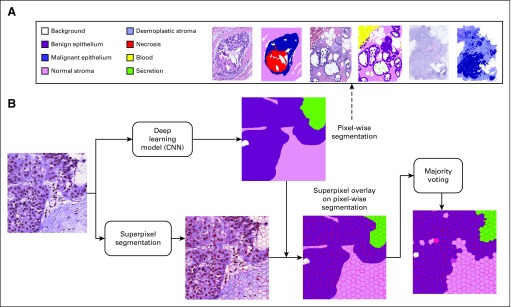
(A) Tissue labels agreed on by pathologists to use in semantic segmentation. Three example patients from the data set are shown; to the right of each patient is a label image with the pixels annotated by a pathologist. (B) Flow diagram. Tissue segmentation has 2 parts: (1) a convolutional neural network (CNN) is trained from expert ground truth data to classify tissues at the pixel level into histopathologic classes, and (2) images are broken into superpixels, which are small, compact areas of the image that have similar color and texture within their boundaries; then, the pixel classification is transferred to the superpixels by assigning each superpixel the class label of the majority of its pixels.

Tissue segmentation has 2 parts: (1) a CNN is trained from expert ground truth data to classify tissues at the pixel level into histopathologic classes, and (2) images are broken into superpixels, which are small, compact areas of the image that have similar color and texture; then, each superpixel is assigned the class label of the majority of its pixels (Fig 2). The superpixel labels, which are the 8 identifiable tissue classes given in the Overview, will be used by the diagnostic classifiers. Figure 2 shows these classes in our ground truth ROI images.

The module SuperpixelSegmentation segments images into nonoverlapping superpixels, using a standard simple linear iterative clustering algorithm, which clusters neighboring pixels based on L, a, b values of the CIELAB color space.^3^ Superpixel segmentation is a traditional unsupervised feature-based segmentation method originally developed by Ren and Malik.^15^

CNNs are powerful tools for supervised semantic segmentation.^16-18^ To better fit the whole-slide image problem, we developed a U-Net style encoder-decoder architecture^19^ that uses modules from the ESPNet architecture developed by Mehta et al^20,21^ for better encoding spatial representations and with several modifications to conventional models: (1) an input-aware encoding block, (2) a new dense connection pattern between encoder and decoder, and (3) a combination of contextual features from multiple decoding paths.^3,20,21^

**FIG 3.**
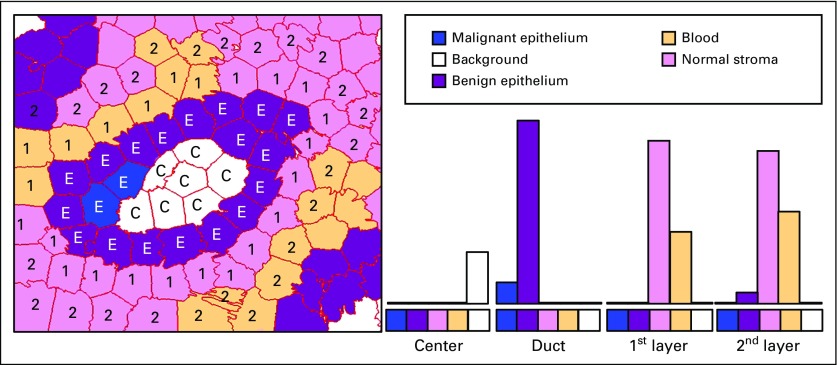
A region is shown with labeled superpixels of 5 different categories. Four different layers are shown in histograms: (C) the duct, (E) the area inside the duct, and 2 outer layers (Nos. 1 and 2 are used to show outer layers 1 and 2). Colors indicate the 5 categories: white, background; blue, malignant epithelium; purple, benign epithelium; pink, normal stroma; and orange, desmoplastic stroma.

In the PixelClassification module, semantic segmentation of biologically meaningful tissues is performed as a multiclass classification of individual pixels using the modified ESPNet-based encoder-decoder model. Our CNN model was trained on size 384 × 384 patches from 58 ROIs fully annotated by an experienced pathologist. Applying this trained CNN classifier on any given ROI yields a labeled image where the tissue types are represented by different integer labels and can be visualized in different colors: 0, Background (white); 2, Benign Epithelium (magenta); 3, Malignant Epithelium (blue); 4, Normal Stroma (pink); 5, Desmoplastic Stroma (violet); 6, Secretion (green); 7, Blood (yellow); 8, Necrosis (red). The next module, SuperpixelClassification, classifies each superpixel with the class of the majority of its pixels. Figure 3 shows an example of labeled superpixels surrounding a duct.

### Diagnosis

Starting with a labeled ROI image, our diagnostic classifiers use the extracted tissue or structure features to produce 1 of 4 diagnoses from benign, atypia, DCIS, and invasive cancer. The modules included in the diagnosis package are as follows: (1) the SuperpixelFrequency module that constructs a superpixel label frequency histogram, (2) the SuperpixelCo-occurrence module that constructs a superpixel label co-occurrence histogram, (3) a MidLevelFeatureClassifier model trained from the combined histograms of superpixel frequency and co-occurrence that assigns diagnostic classes to new ROIs, (4) a StructureFeature module that constructs feature vectors that describe structures important for diagnosis, and (5) a StructureFeatureClassifier model trained from structure feature histograms that assign diagnostic classes to new ROIs. These modules come from the algorithms developed by Mercan et al.^4^

**FIG 4.**
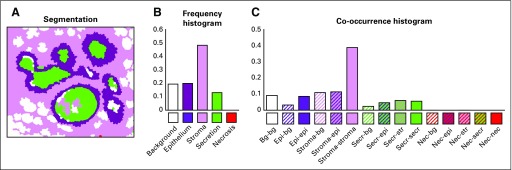
(A) Segmentation, (B) frequency histogram, and (C) co-occurrence histogram from a segmented region of interest. bg, background; epi, epithelium; nec, necrosis; secr, secretion; str, stroma.

The SuperpixelFrequency module takes in a labeled ROI as shown in Figure 3 and produces an 8-bin histogram of the frequencies of each of the 8 histopathologic tissue labels in the ROI. The SuperpixelCo-occurrence module produces an 8- × 8-bin histogram, in which the *(i, j)^th^* bin contains the frequency of label *i* appearing adjacent to label *j* in the ROI. Superpixel co-occurrence can show the frequency of contact between superpixels of different tissue types. Figure 4 shows an example of a segmented tissue map and its superpixel label frequency histogram and superpixel label co-occurrence histogram.

The MidLevelFeatureClassifier module is a 4-class classifier trained from the feature vectors computed by the SuperpixelFrequency and SuperPixelCo-occurrence modules on 428 ROIs in the full data set. Given the frequency and co-occurrence histograms of segmented ROIs, the classifier assigns 1 of the 4 diagnosis labels: benign, atypia, DCIS, and invasive.

**FIG 5.**
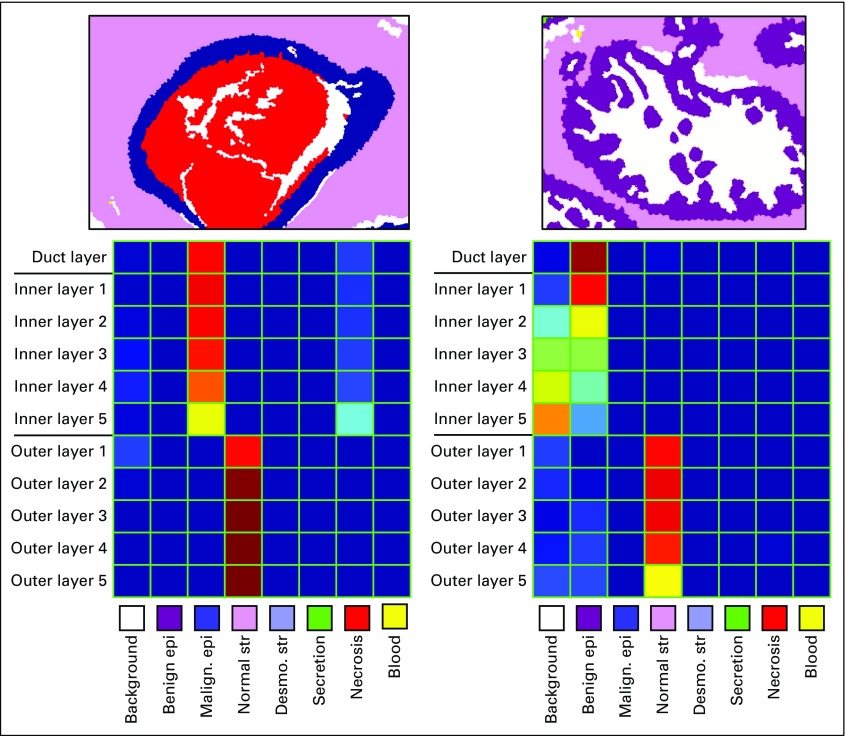
An example heatmap of structure features extracted. The top row shows the result of tissue label segmentation. The columns represent the location of the layer, and the rows represent the tissue types presented, with relative frequency represented by color: blue, lower frequency; red, higher frequency. Desmo., desmoplasia; epi, epithelium; malign., malignant; str, stroma.

The StructureFeature module was developed with the advice of expert pathologists to describe the structures they pay close attention to during diagnosis, in particular, structural changes surrounding a duct. Using the semantic segmentation result, objects of interests or central structures (ducts) are identified, and the feature extraction process is applied: 1 superpixel–wide layers of superpixels toward the inside of the object and toward the outside. For each layer, a frequency histogram of tissue labels of superpixels within each such layer is constructed. Figures 3 and 5 show examples of structure feature extraction from different ROIs.

The StructureFeatureClassifier module is a 4-class classifier trained on the structure features obtained from applying the StructureFeature module to 428 ROIs in the full data set. Given the structure feature histograms as shown in Figures 3 and 5 of labeled ROIs, the classifier assigns 1 of the 4 diagnoses labels: benign, atypia, DCIS, and invasive breast cancer.

## RESULTS

**FIG 6.**
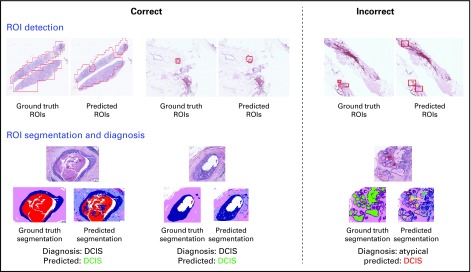
Example results. For region of interest (ROI) detection (top), the results on the left are examples of where the predicted ROIs are similar to the ground truth regions; the result on the right is an example of where they are incorrect or incomplete. ROIs are detected by the ROIDetection module. For ROI segmentation and diagnosis (bottom), the results on the left are examples of where the segmented ROIs are similar to the ground truth segmentations; the example on the right shows a poor segmentation and incorrect diagnosis result. DCIS, ductal carcinoma in situ.

This software package comes from research work whose code was written in multiple languages and packages, including C++, MATLAB, and Torch. All of the software is being converted to Python modules to produce a uniform machine-learning-based package. The modules described in this article are complete, with the exception of the ongoing work on the StructureFeature extractor and classifier; they can be downloaded from https://github.com/cancertech/cancer_diagnosis. Instructions for using the package are given on our Web page (https://cancertech.cs.washington.edu/), which has tabs for downloading the whole package as a zip file, installation of required software including Anaconda/Python, and a tutorial that takes the user through the process of running the package on an image through the whole pipeline. Figure 6 shows sample results, which are more comprehensive than the simple example in the tutorial. At the top are 3 examples of ROI detection in which the left 2 approximately agree with ROIs labeled by pathologists and the right one has an extra ROI that was not in the ground truth; we attribute this to the denseness of cells and duct-like structures. The accuracy of the algorithm was reported in Mercan et al^2^ as 0.72. Below are results of the SuperpixelClassification and MidLevelFeatureClassifier diagnosis modules in which the leftmost 2 examples show segmentations similar to the ground truth, whereas the example on the right shows a hard case with poor segmentation and incorrect diagnosis.

This segmentation software comes from the work of Mehta et al^3^ that performs both semantic segmentation and diagnosis and was evaluated on 428 ROIs with diagnostic labels and 58 with hand-marked tissue labels. In this work, the segmentor obtained a 0.44 mIOU (mean intersection over union of the pixel regions) score, which was enough to obtain a 0.63 diagnostic accuracy for the 4-class problem compared with 44 pathologists in the study who obtained 0.70 accuracy. The 4-class problem is quite difficult, and the later work of Mercan et al^4^ obtained higher accuracies by breaking the problem into binary problems in a tree structure and using additional features. The algorithms we have implemented are part of the Mercan et al^4^ work. Our midlevel feature classifier obtains approximately the same accuracies reported in that work (0.94 in invasive *v* others; 0.70 for benign *v* DCIS and atypia; 0.83 for DCIS *v* atypia). The 0.83 accuracy for DCIS versus atypia was higher than the 0.80 accuracy of pathologists in our study.

## DISCUSSION

Our scientific methodology was produced by a multidisciplinary team consisting of world experts in epidemiology, breast pathology, and computer vision/machine learning. The current classifiers are all trained on a specific and well-developed breast biopsy data set. Although they are meant to be used on breast cancer whole-slide images, we plan to make the untrained versions of the classifiers available for researchers to use on their own data sets, with an extension to other cancers in the near future. The methodology has been shown to be transferable to our current work on a melanoma biopsy set. Pathologists’ annotations and tracking data can be used to train a (new) ROI classifier. An expert pathologist can mark the borders of different tissues on ROIs (ongoing), and then the Tissue Segmentation module can work in the same way. The labels, of course, are different for different tissue types. Once the semantic segmentation is completed, the diagnostic classification through label frequency and co-occurrence can run as described. However, the structure feature was meant for ducts or other small areas, such as glands, that would not apply to all cancers.

The package was initially intended for cancer researchers but shows functionality for practitioners. In both cases, a suitable interface to improve the user experience will be necessary. In its current state, the software must be downloaded from GitHub, and the modules are run by the user because some of them extract features from large images. The long-term plan is to make the software available through the PIIP package.^5^ PIIP is an NCI/NIH-sponsored project intended for managing, annotating, sharing, and quantitatively analyzing digital pathology imaging data that expands on an existing, freely available pathology image viewer, Sedeen. We chose the PIIP/Sedeen platform because it is readily available, is easy to use, and provides excellent annotation tools for training deep-learning classifiers, Sedeen will read in a whole-slide image and pass it to the tools of our package, which will reside in PIIP. Because Sedeen is Windows based, our first software is also Windows based, but we will provide Linux and MacOS as well in the near future. SVS format will be supported on Linux and MacOS after installation of openslide (see https://openslide.org/download/ for installation details).

Software development for computer-aided cancer diagnosis is one of the most difficult problems in cancer informatics research and requires a dynamic and strategic research team for our NIH/NCI-sponsored project, U01 CA231782. The resulting design for AI-assisted diagnosis includes ROI finding, tissue segmentation into histopathologic classes using a state-of-the-art deep-learning scheme and classification into multiple diagnosis categories using hand-crafted features and SVMss. Our new tools show applicability and adaptability to a range of cancer researchers beyond our initial research into breast cancer data sets and eventually to practicing pathologists, from residents who are learning diagnostic skills to experts who want a quick second opinion on patients for which additional advice is desired.
